# Herpes Simplex Virus 1 Spread in Oligodendrocytic Cells Is Highly Dependent on MAL Proteolipid

**DOI:** 10.1128/JVI.01739-19

**Published:** 2020-01-31

**Authors:** José Antonio López-Guerrero, Carmen de la Nuez, Beatriz Praena, Enrique Sánchez-León, Claude Krummenacher, Raquel Bello-Morales

**Affiliations:** a Universidad Autónoma de Madrid, Departamento de Biología Molecular, Madrid, Spain; b Centro de Biología Molecular Severo Ochoa, CSIC-UAM, Madrid, Spain; c Department of Biological Sciences and Department of Molecular and Cellular Biosciences, Rowan University, Glassboro New Jersey, USA; Northwestern University

**Keywords:** HSV-1, MAL, oligodendrocytes, myelin, cell-to-cell spread, viral spread

## Abstract

Myelin and lymphocyte protein (MAL) is a tetraspan integral membrane protein that resides in detergent-insoluble membrane fractions enriched in condensed membranes. MAL is expressed in oligodendrocytes, in Schwann cells, where it is essential for the stability of myelin, and at the apical membrane of epithelial cells, where it has a critical role in transport. In T lymphocytes, MAL is found at the immunological synapse and plays a crucial role in exosome secretion. However, no involvement of MAL in viral infections has been reported so far. Here, we show that herpes simplex virus 1 (HSV-1) virions travel in association with MAL-positive structures to reach the end of cellular processes, which contact uninfected oligodendrocytes. Importantly, the depletion of MAL led to a significant decrease in infection, with a drastic reduction in the number of lytic plaques in MAL-silenced cells. These results suggest a significant role for MAL in viral spread at cell contacts. The participation of MAL in the cell-to-cell spread of HSV-1 may shed light on the involvement of proteolipids in this process.

**IMPORTANCE** Herpes simplex virus 1 (HSV-1) is a neurotropic pathogen that can infect many types of cells and establish latent infections in neurons. HSV-1 may spread from infected to uninfected cells by two main routes: by cell-free virus or by cell-to-cell spread. In the first case, virions exit into the extracellular space and then infect another cell from the outside. In the second case, viral transmission occurs through cell-to-cell contacts via a mechanism that is still poorly understood. A third mode of spread, using extracellular vesicles, also exists. In this study, we demonstrate the important role for a myelin protein, myelin and lymphocyte protein (MAL), in the process of cell-to-cell viral spread in oligodendrocytes. We show that MAL is involved in trafficking of virions along cell processes and that MAL depletion produces a significant alteration in the viral cycle, which reduces cell-to cell spread of HSV-1.

## Abstract (precis)

Herpes simplex virus 1 (HSV-1) is a neurotropic pathogen that can infect many types of cells and establish latent infections in neurons. HSV-1 may spread from infected to uninfected cells by two main routes: by cell-free virus or by cell-to-cell spread. In the first case, virions exit into the extracellular space and then infect another cell from the outside. In the second case, viral transmission occurs through cell-to-cell contacts via a mechanism that is still poorly understood. A third mode of spread, using extracellular vesicles, also exists. In this study, we demonstrate the important role for a myelin protein, myelin and lymphocyte protein (MAL), in the process of cell-to-cell viral spread in oligodendrocytes. We show that MAL is involved in trafficking of virions along cell processes and that MAL depletion produces a significant alteration in the viral cycle, which reduces cell-to cell spread of HSV-1.

## INTRODUCTION

Myelin and lymphocyte protein (MAL) (1) is the prototypical member of the MAL proteolipids, a protein family sharing structural and biochemical similarities found in detergent-insoluble membranes enriched in glycolipids and cholesterol (2). MAL is a nonglycosylated integral membrane protein of 17 kDa containing four hydrophobic segments that resides in detergent-insoluble membrane fractions enriched in condensed membranes (3–5). Initial studies described MAL as a proteolipid of the detergent-resistant membrane microdomains of T lymphocytes which is associated with glycosylphosphatidylinositol-anchored proteins (4, 6). MAL proteolipids also play a crucial role in exosome secretion by human T cells, a role probably related to its capacity for organizing condensed membrane domains or rafts (7, 8). MAL is also expressed in apical membranes of epithelial cells and has a critical role in normal apical transport. In epithelial Madin-Darby canine kidney (MDCK) cells, MAL is critical for the transport of apical secretory proteins (9) and for the correct condensation of membranes at the ciliary base, which is required for efficient primary cilium extension (10). Suppression of MAL expression in epithelial cells is also linked to several types of cancer, suggesting a function as a tumor suppressor, an antimetastasis factor, and an inhibitor of tumorigenicity through the induction of apoptosis (11–13). In addition, MAL is involved in the correct sorting of influenza virus hemagglutinin in epithelial cells (3).

In the nervous system, MAL is found in oligodendrocytes (OLs) and Schwann cells, where it is predominantly located in compact myelin (14–16). In the central nervous system (CNS), this tetraspan proteolipid plays an essential role in the stability of myelin (15) and regulates the distribution of PLP, the main myelin protein, into distinct membrane microdomains, allowing its lateral diffusion from the cell membrane to the myelin once the myelin sheath has been assembled (17). MAL-deficient mice showed defects in the maintenance of myelinated CNS axons, indicating anomalous protein trafficking and/or sorting in OLs lacking MAL (15, 18). On the other hand, MAL overexpression delayed maturation of nonmyelinating and myelinating Schwann cells (19) and aberrant myelin formation in the CNS, as well as formation of large cysts in kidneys, indicating that increased expression of MAL is deleterious to membranous structures in the affected tissues. Thus, a tight control of MAL expression is required for the function of myelinating and epithelial cells (20). In addition, MAL seems to participate in several pathophysiologic processes in the CNS. For instance, MAL plays a crucial role in the neuronal apoptosis after spinal cord injury in rats (21, 22). MAL is required for cell binding and cytotoxicity of Clostridium perfringens epsilon toxin (ETX), a potent toxin which causes blood-brain barrier dysfunction and white matter injury and which has been involved in multiple sclerosis (MS) etiology (23, 24).

No effect of MAL on viral infections has been reported so far. In previous studies, we noted a partial colocalization of herpes simplex virus 1 (HSV-1) particles with exogenous MAL in vesicles located at the end of cellular processes in OLs (25). We also reported the role of microvesicles in HSV-1 transmission between OLs (26). Given the involvement of MAL in exosome secretion (7), we investigated whether viral particles might be travelling into MAL-positive vesicles during viral spread (25). We used a short hairpin RNA to produce a stable MAL-silenced human oligodendroglioma (HOG) cell line and demonstrated a functional role of MAL in HSV-1 spread. MAL silencing led to a drastic decrease in plaque formation in HOG cells. Imunogold-labeling electron microscopy (EM), fluorescence video microscopy, and immunofluorescence microscopy showed an association of viral capsids and MAL-positive structures in these cells. Trafficking of virions with MAL vesicles along cellular processes was associated with virus spread. Altogether, these data show and explain for the first time the significant influence of MAL proteolipid on the viral cycle of HSV-1 in oligodendrocytic cells. Further studies will have to confirm whether these results can be extrapolated to other cell types.

## RESULTS

### Overexpression of exogenous MAL in HOG cells.

We previously observed colocalization of virions with MAL-positive vesicles in HOG cells (25). Since there is only a low level of MAL proteolipid expression in these cells, and to improve the detection of MAL and perform a kinetic analysis of trafficking in live cells, we used a previously described (27) HOG cell line stably transfected with MAL-diHcRed, a construction consisting of MAL protein tagged with diHcRed, a dimeric red fluorescent protein (28, 29). To study the distribution of MAL-diHcRed in mock and HSV-1-infected HOG cells, we performed immunofluorescence and EM analysis.

**FIG 1:**
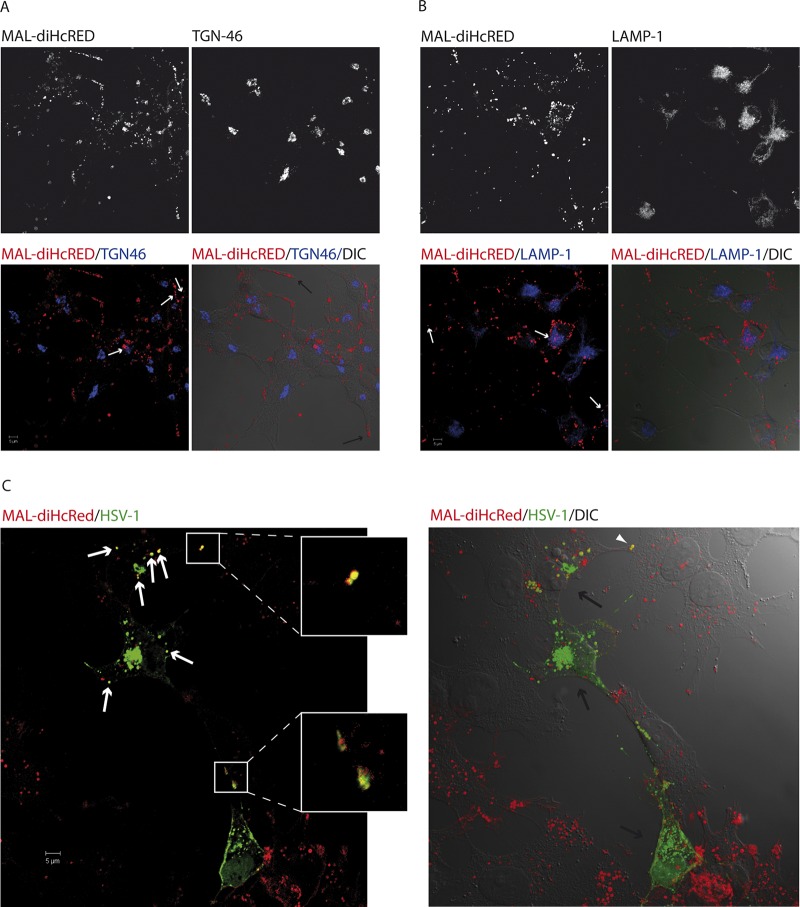
Overexpression of exogenous MAL in HOG cells and infection with HSV-1. HOG MAL-diHcRed cells cultured on glass coverslips were fixed and processed for immunofluorescence and incubated with a rabbit anti-TGN46 antibody (A) and a sheep anti-LAMP-1 antibody (B). Alexa-647 donkey anti-rabbit and anti-sheep secondary antibodies were used, respectively, to detect the primary antibodies. (A) Distribution of exogenous MAL at the plasma membrane and also in vesicular structures located in the cytoplasmic vesicular structures concentrated at the ends of the processes (black arrows). Exogenous MAL showed a partial colocalization with the TGN (white arrows). (B) In addition, exogenous MAL showed partial colocalization with the endosomal/lysosomal membrane protein LAMP-1 (white arrows). (C) HOG MAL-diHcRed cells were infected with HSV-1 at an MOI of 0.5. At 24 h p.i., cells were fixed and processed for immunofluorescence microscopy and incubated with a rabbit anti-HSV-1 antibody followed by an Alexa-488 donkey anti-rabbit secondary antibody. Images show the presence of infected (green signal, black arrows) and uninfected cells. A confocal 0.6 μm slice shows the presence of exogenous MAL-positive vesicles colocalizing with viral particles (arrowheads; magnified in the white square). A DIC image was merged with the fluorescence projection of the confocal planes to better show the location of MAL-diHcRed-positive vesicles containing virions at the end of the processes contacting adjacent uninfected cells (arrowhead).

HOG MAL-diHcRed cells cultured on glass coverslips were fixed and processed for immunofluorescence as described in Materials and Methods. In noninfected cells, MAL-diHcRed was located at the plasma membrane and in cytoplasmic vesicular structures which were concentrated near the ends of processes extended from the cell surface (Fig. 1A). We also observed a partial colocalization of MAL-diHcRed with TGN46, a marker of the trans-Golgi network (TGN) (Fig. 1A) and with the endosomal-lysosomal membrane protein LAMP-1 (Fig. 1B). We then infected HOG MAL-diHcRed cells with HSV-1 at a multiplicity of infection (MOI) of 0.5. At 24 h postinfection (p.i.), the distribution of exogenous MAL-positive vesicles was not altered. However, several MAL-diHcRed-positive vesicles colocalized with anti-HSV staining (Fig. 1C). Interestingly, MAL-positive vesicles containing virions were located at the end of the processes which contacted adjacent uninfected cells (Fig. 1C). This observation supports the hypothesis that MAL-positive vesicles might be carriers of virions toward contacts with uninfected cells.

**FIG 2:**
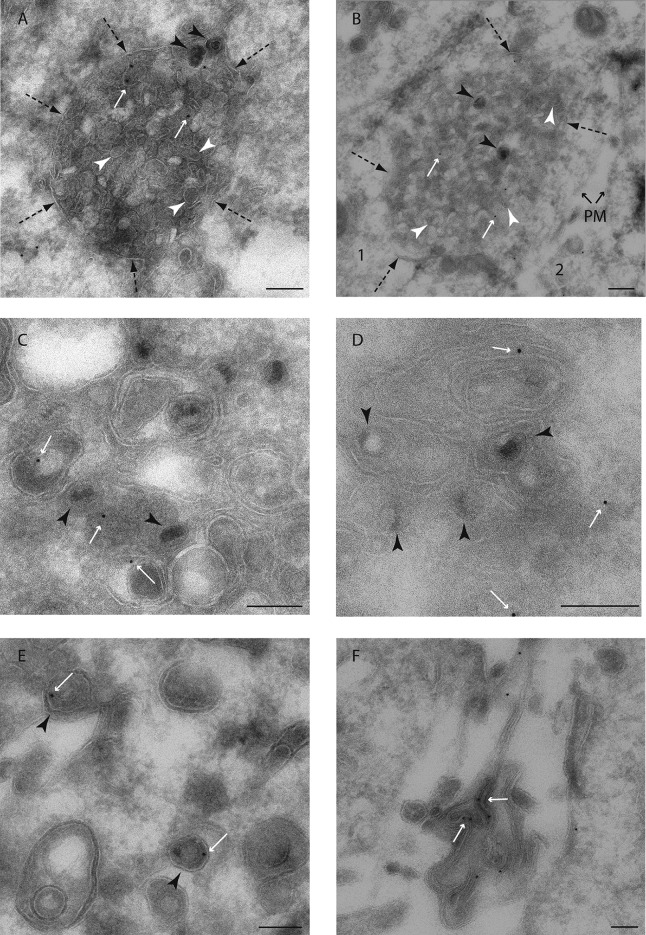
EM of HOG-MAL-diHcRed-infected cells. HOG MAL-diHcRed cells were mock infected or infected with HSV-1 at an MOI of 1 and incubated for 24 h at 37°C. Then cells were fixed and processed for observation using immunoelectron microscopy. Cryosections were stained with a rabbit anti-RCFP antibody. Primary antibody was detected with 15 nm anti-rabbit-gold (white arrows). EM images revealed the presence of MAL-diHcRed labeling in MVBs containing virions (A and B). The MVB (B, cell 1) is located in the close proximity of another cell (cell 2), thus allowing a feasible release of the viral particles tightly close to the neighboring cell after fusion with the plasma membrane (PM). MAL-diHcRed-positive vesicles were observed also in regions of viral membrane wrapping (C and D) and enveloped virions (E). Finally, we detected MAL-diHcRed in multilamellar structures located in the extracellular space (F). Black arrowheads point to both enveloped and unenveloped HSV-1 virions. Dashed arrows point to the limiting membrane of the MVBs, whereas the white arrowheads point to intraluminal vesicles. Scale bar, 200 nm.

In order to better characterize these exogenous MAL-positive structures associated with virions, we used immuno-electron microscopy. In infected HOG cells, immunostaining of exogenous MAL-diHcRed showed an association with multivesicular bodies (MVBs) containing virions (Fig. 2A and B). The presence of enveloped viruses in MAL-positive MVBs near the plasma membrane (Fig. 2B) is consistent with the immunofluorescence data (Fig. 1). Fusion of MVBs with the plasma membrane leading to the release of intraluminal vesicles and virions into the intercellular space might place the viral particles in close contact with neighboring cells, thereby favoring viral spread. In addition, MAL-diHcRed-positive vesicles were observed in regions of viral envelopment (Fig. 2C and D), and in addition, MAL-diHcRed signal was observed in enveloped virions (Fig. 2E). Finally, we detected MAL-diHcRed in multilamellar structures apparently located in the extracellular space (Fig. 2F). Similar multilamellar structures, positive for myelin proteins, have been observed before along the cell body and processes of cultured OLs (30). Altogether, these immune-EM data confirm the association of virions with MAL-positive MVBs.

### Fluorescence video microscopy of HOG-MAL-diHcRed infected cells.

**FIG 3:**
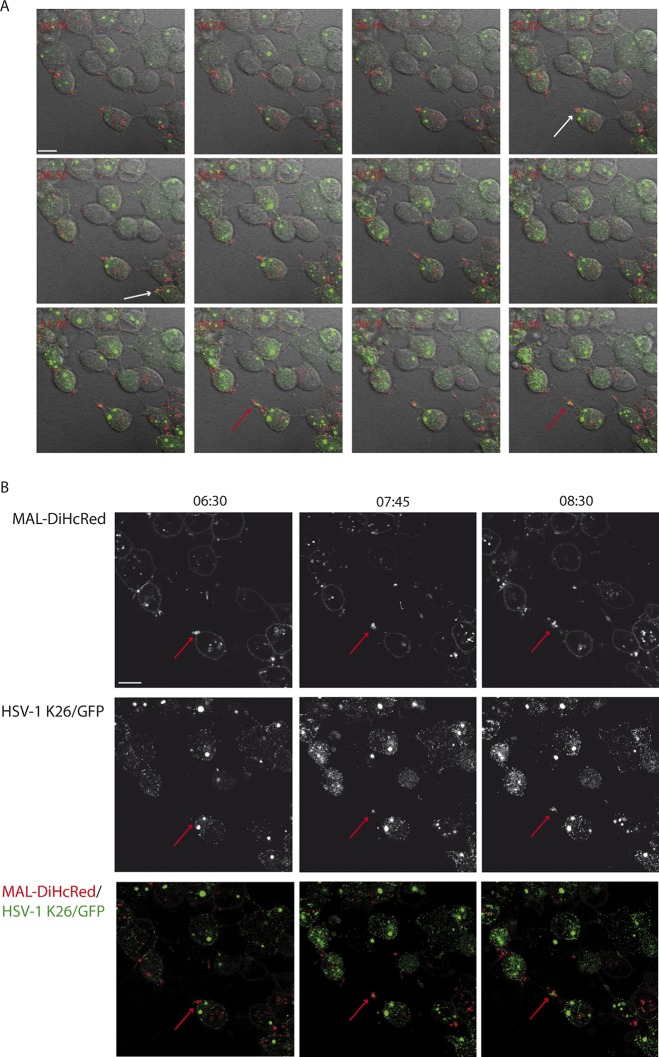
Colocalization of exogenous MAL in HSV-1 K26GFP-infected cells. Cells plated in 35-mm glass-bottom dishes were infected with HSV-1 K26GFP at an MOI of 1. Six hours later we performed video microscopy recording green (GFP, virus), red (exogenous MAL), and DIC signals. (A) Time-lapse images show MAL-diHcRed accumulation in the poles of balloon-infected cells (white arrows). Gradually, virions gathered near the cell poles and colocalized with MAL to finally traffic together along the processes (red arrows). Images correspond to confocal slices of infected cells. (B) For better visualization of signals, images of red and green channels are shown independently at various times after the beginning of the recording (6:30, 7:45, and 8:30 h). At 6:30 h, the accumulation of MAL in the cell pole (red arrows) was observed. At 7:45 and 8:30 h the partial colocalization of MAL with virions is patent (red arrows). Scale bar, 10 μm.

**FIG 4:**
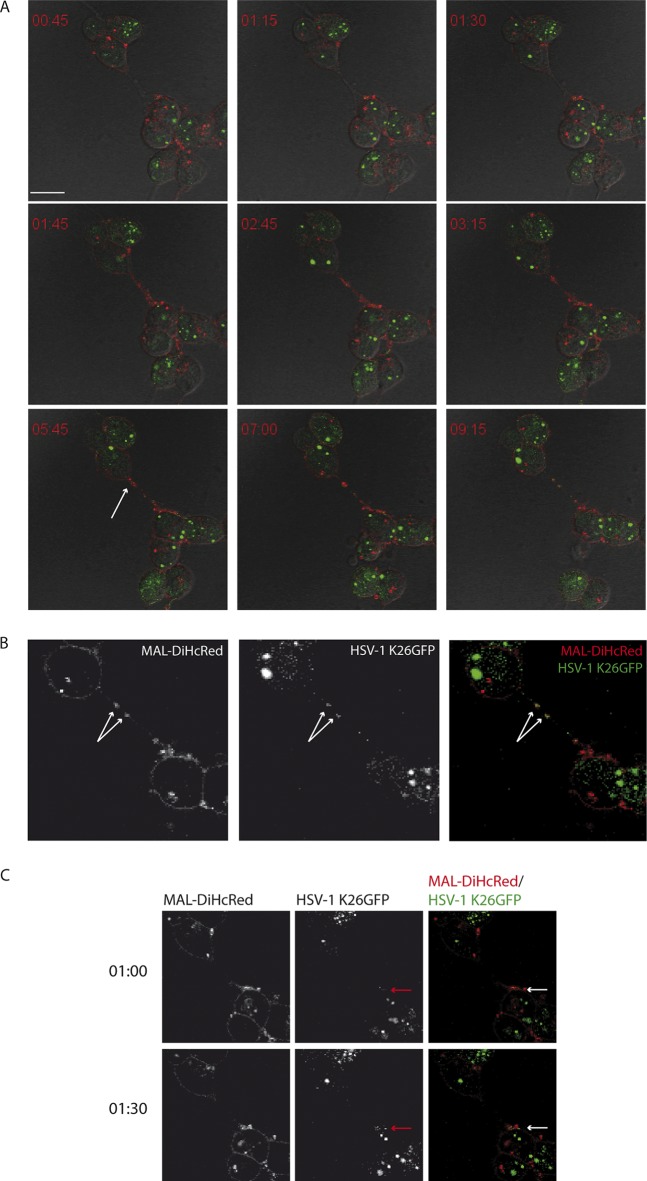
Traffic of exogenous MAL-associated virus from cell to cell. HOG-MAL-diHcRed cells plated in 35-mm glass-bottom dishes were infected with HSV-1 K26GFP at an MOI of 1. Ten hours later we performed video microscopy recording green (GFP, virus), red (exogenous MAL), and DIC signals. (A) In these time-lapse images, the traffic of MAL from one cell to another through the processes is patent. After 5:45 h a cluster of MAL and virions reached the neighboring cell (white arrow). (B) For better visualization of signals, red and green channels are shown separately. At 9:15 h, the partial colocalization of MAL with virions is manifest (white arrows). (C) Images show the gradual accumulation of virions in the cell pole (GFP signal) and its colocalization with MAL. From 1:00 to 1:30 h, a clear increment in the number of virions near the cell pole (red arrows) is manifest. Scale bar, 10 μm.

**FIG 5:**
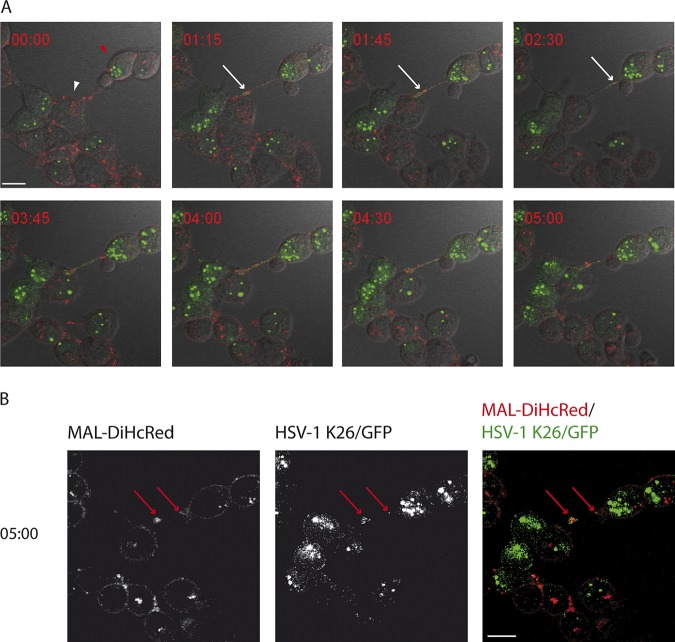
Traffic of exogenous MAL-associated virus to uninfected cells. HOG-MAL-diHcRed cells plated in 35-mm glass-bottom dishes were infected with HSV-1 K26GFP at an MOI of 1. Ten hours later we performed video microscopy recording green (GFP, virus), red (exogenous MAL), and DIC signals. (A) Time-lapse images show the traffic of virions associated with MAL from one cell (red arrowhead) to an uninfected target cell (white arrowhead) through a cell process (white arrows). (B) For better visualization of signals, red and green channels are shown separately. At 5:00 h (10 h p.i.) the partial colocalization of MAL with virions is clearly visible (red arrows). Scale bar, 10 μm.

To study the dynamics of HSV-1 infection in live HOG-MAL-diHcRed cells, we performed time-lapse fluorescence microscopy using the fluorescent HSV-1 recombinant virus K26GFP (25, 31). We infected HOG-MAL-diHcRed cells with HSV-1 K26GFP at an MOI of 1 and recorded green (viral capsids), red (MAL-diHcRed), and DIC signals. Our results showed the association of exogenous MAL with virus in long processes extending from the cell surface. (Fig. 3; see Video S1 in the supplemental material). HOG cells infected with HSV-1 displayed a characteristic ballooning as a part of the cytopathic effect (CPE) caused by the infection. In these balloon cells, MAL accumulated preferentially at specific peripheral locations (poles) (Fig. 3A, white arrows). As infection progressed, green fluorescent protein (GFP) capsids accumulated in these MAL-positive structures. GFP capsids and MAL-diHcRed continued to colocalize and moved together along cellular processes toward the tip of these processes (Fig. 3A, red arrows). Fluorescent images in Fig. 4 and Video S2 show that virus trafficking with MAL-diHcRed along the processes reached an adjacent cell (Fig. 4A). The colocalization of virions with MAL was maintained throughout (Fig. 4B). These data indicate that virions traffic in MAL-positive vesicles along cell processes in HOG cells and are directed toward adjacent cells. The trafficking of virions associated with MAL-positive vesicles toward a noninfected cell appears to spread infection to the adjacent cell (Fig. 5; Video S3). Thus, a cell that initially lacks viral GFP signal (Fig. 5A, arrowhead), becomes gradually infected—as seen by the increase in GFP—after MAL-diHcRed and GFP-positive structures reach it through the processes. Partial colocalization of MAL-diHcRed and GFP is patent along the cell processes (Fig. 5B). It is, however, not possible to rule out that virions from adjacent cells or free virions could have infected the target cell. Overall, these data are consistent with virions being trafficked with MAL to the tip of cellular processes to later infect adjacent cells.

### Generation and infection of MAL-silenced HOG cells.

**FIG 6:**
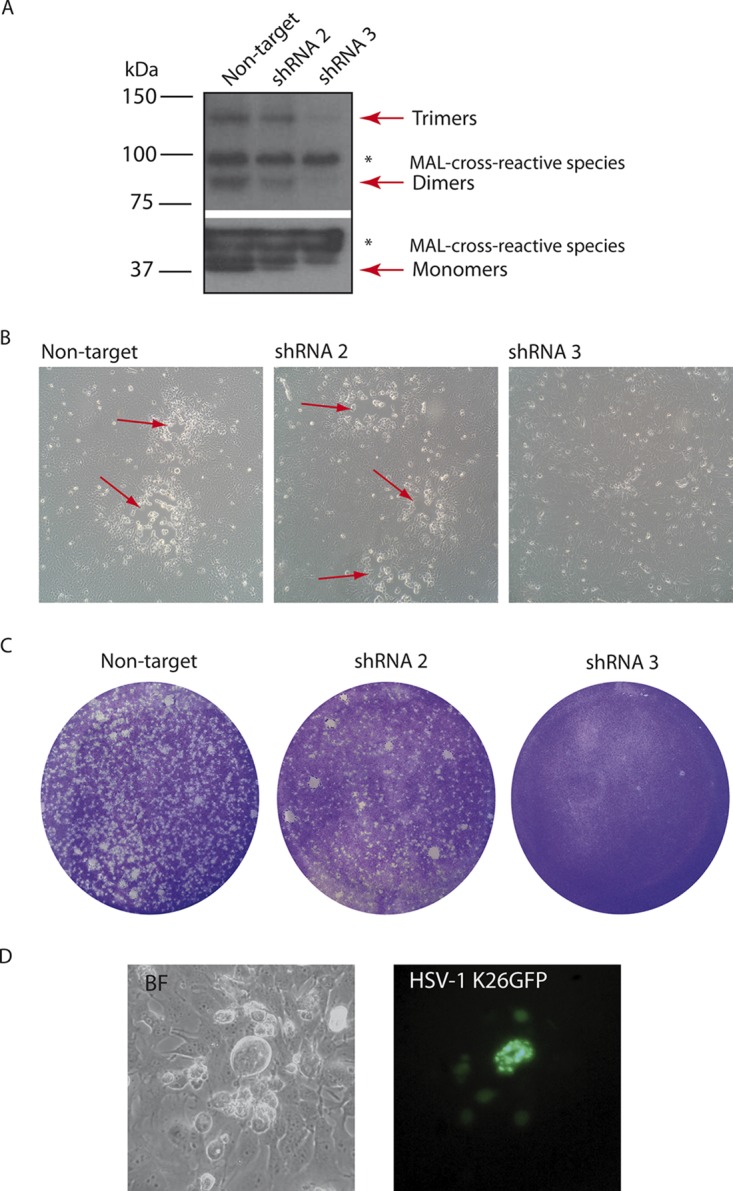
Generation and infection of MAL-silenced HOG cells. (A) HOG-MAL-diHcRed cells were transfected with MAL-silencing shRNA-2 or shRNA-3, and an shRNA nontarget control. Equal numbers of cells were lysed, analyzed using a Bradford assay to equalize the protein load, subjected to SDS-PAGE, and analyzed using immunoblotting with anti-MAL antibody 6D9. Plasmid shRNA-3 induced an efficient knockdown, while shRNA-2 elicited a weaker effect. (A) The nonspecific cross-reacting bands (asterisks) serve as protein load controls. (B) Bright-field images show the effect of MAL knockdown on the plaque formation in live cells, with a drastic decrease in the number of plaques (red arrows) in shRNA-3 silenced cells compared to shRNA-2 cells and the nontarget control. (C) Confluent monolayers of cells plated in 6-well tissue culture dishes were infected with HSV-1 and processed for plaque assay. Images show a drastic reduction in the number of plaques in shRNA-3 and shRNA-2 silenced cells compared to the nontarget control. (D) Fluorescence microscopy reveals that GFP signal was present in shRNA-3 silenced cells infected with HSV-1 K26GFP 24 h p.i.

To determine whether MAL plays an active role in HSV-1 spread from HOG cells, we carried out functional studies using MAL short hairpin RNA (shRNA) knockdown. To generate stably silenced cell lines, HOG-MAL-diHcRed cells were transfected with two plasmids expressing MAL shRNAs. One of them, shRNA-3, induced an efficient knockdown of MAL, while a second plasmid, shRNA-2, elicited a weaker effect (Fig. 6A). We analyzed the silencing by immunoblotting using equal amounts of proteins from total lysates of the various cell lines. To corroborate the equal protein load, we used the nonspecific bands from proteins cross-reacting with the MAL antibody 6D9, which have been reported previously (32). Immunoblotting showed the efficient knockdown of MAL with shRNA-3, as seen for MAL-monomers, dimers, and trimers (Fig. 6A). Although MAL does not form large oligomers, the presence of dimers and trimers has also been reported before (33). ShRNA-2 silencing was less efficient (Fig. 6A), but we nevertheless used these cells to compare the effects of different degrees of silencing. We needed to use HOG MAL-diHcRed cells in these experiments since only these cells allowed us to assess the efficacy of MAL silencing. The small amount of MAL in normal HOG cells was not reliably detectable by Western blotting, and thus silencing could not be monitored adequately.

After confirmation of MAL depletion, we analyzed the susceptibility of MAL-silenced cells to HSV-1 infection. First, we studied the effect of MAL knockdown on cytopathic effect (CPE) and plaque formation. The presence of CPE and lytic plaques decreased drastically in shRNA-3 silenced cells compared to shRNA-2 silenced cells and control cells with nontarget shRNA (Fig. 6B).

Similarly, a plaque assay showed a drastic decrease of the number of plaques in silenced shRNA-3 cells compared to shRNA-2 and nontarget control cells (Fig. 6C). We noted that shRNA-3 silenced HOG MAL-diHcRed cells showed decreased plaque formation compared to natural HOG cells. This suggests that our knockdown approach also led to silencing the already low level of endogenous MAL (data not shown). These data indicate that MAL plays an essential role in the viral cycle. However, when we infected shRNA-3 MAL-silenced cells with HSV-1 K26GFP, we observed that these cells efficiently produced green VP26-GFP capsid protein (Fig. 6D). This observation suggests that viral entry and replication were not affected by MAL depletion. However, the absence of lytic plaques supports an essential role for MAL in virus spread.

### Role of MAL in the viral cycle.

**FIG 7:**
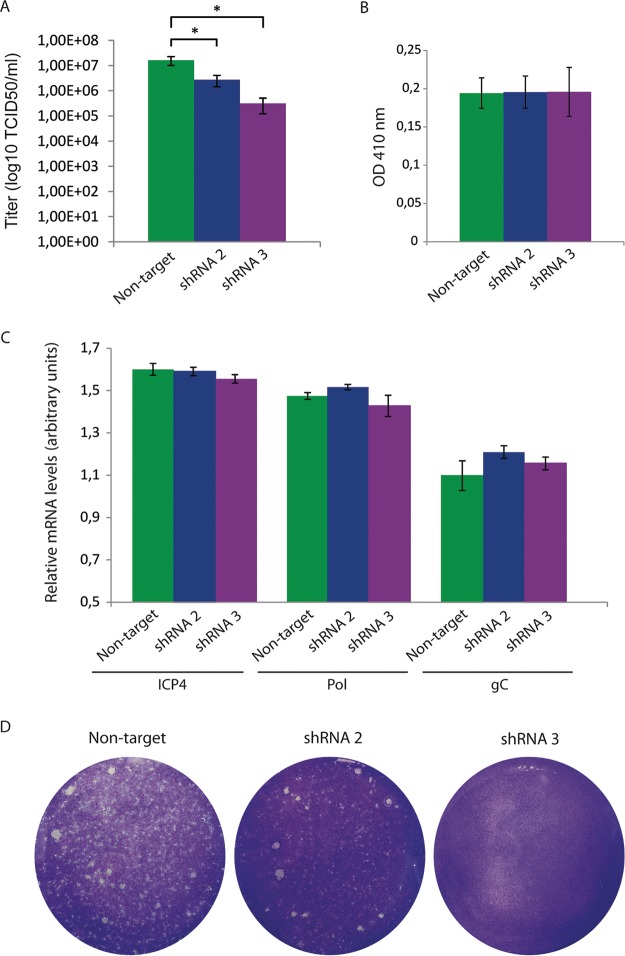
Effect of MAL silencing on the HSV-1 viral cycle. (A) MAL-silenced cells were infected with HSV-1, and 24 h p.i., the viral titer was quantified using an endpoint dilution assay determining the TCID_50_ in Vero cells. Viral production in shRNA-3 silenced cells showed a significant decrease compared to shRNA-2 and the nontarget control (*, *P* < 0.05). (B) Infection with the recombinant HSV-1 gL86, which expresses β-galactosidase upon entry into cells. Absorbance at 410 nm was used to monitor beta-galactosidase activity. No significant change was observed in shRNA-3 cells compared to the nontarget control. (C) RT-qPCR of mRNAs. Bars represent relative mRNA levels corresponding to ICP4 (extracted at 3 h p.i.), viral polymerase (extracted at 6 h p.i.), and gC (extracted at 9 h p.i.) from HSV-1-infected nontarget, shRNA-2, and shRNA-3 cells. (D) Confluent monolayers of cells plated in 6-well tissue culture dishes were infected with HSV-1 in the presence of human serum 1% and processed for plaque assay.

To analyze whether MAL depletion altered viral production, we quantified the viral titer at 24 h p.i. by an endpoint dilution assay determining the 50% tissue culture infective dose (TCID_50_) in Vero cells. Viral production corresponding to cell-associated virus at 24 h p.i. decreased by about 2 orders of magnitude in shRNA-3 silenced cells compared to nontarget control ShRNA-2 silenced cells, which showed a less pronounced decrease, correlating with a less efficient MAL silencing (Fig. 7A). Next, we analyzed the effect of MAL depletion on viral entry by infecting cells with the recombinant HSV-1 gL86 virus, which expresses beta-galactosidase upon entry into cells. Figure 7B shows that beta-galactosidase activity was not significantly reduced in shRNA-3 cells compared to nontarget control cells. In HSV-1 gL86, beta-galactosidase expression is driven by the gL late promoter (34). Therefore, MAL depletion did not affect entry and late expression of beta-galactosidase. To further confirm that MAL plays a role in the final part of the viral cycle, we measured mRNA expression of representative immediate early (ICP4), early (polymerase), and late (gC) genes (Fig. 7C). Quantitative reverse transcription PCR (RT-PCR) analyses showed that expression of these genes was not significantly changed in MAL-depleted cells (Fig. 7C). These data are consistent with MAL playing a role in the spread of HSV-1 from HOG cells.

To investigate how MAL silencing affected viral spread, infected cells were cultured in the presence of 1% pooled human serum to neutralize extracellular virus, thereby restricting plaque formation to direct cell-to-cell transmission. In the presence of anti-HSV human serum, lytic plaques are only due to cell-to-cell spread of virus through cell junctions or other types of tight contacts such as neuronal synapses. Here, we observed that knockdown of MAL decreased the number of plaques in both shRNA-2 and -3 cells compared to nontarget control cells (Fig. 7D). Again, shRNA-3 cells were more dramatically affected than shRNA-2 cells because of the more efficient knockdown of MAL. This finding suggests an effect of MAL depletion on direct cell-to-cell spread. It is noteworthy that in OLs only a small fraction of infectious HSV-1 is released into the extracellular medium as free virions. Indeed, the amount of extracellular HSV-1 released from infected HOG cells 24 h p.i. is more than 1 order of magnitude lower than the amount of cell-associated virus (our unpublished results). Thus, MAL may have more of an influence on viral spread in cells relying on cell-to-cell spread, such as OLs, than other cell types.

## DISCUSSION

HSV-1 may spread from infected to uninfected cells by two main routes, by cell-free virus or by direct cell-to-cell spread. In the first case, virions exit into the extracellular space and then infect another cell from the outside. In direct spread, the viral transmission occurs through cell-to-cell contacts via a mechanism that is still poorly understood. Alternatively, viral spread may occur through extracellular vesicles, which we have shown to occur in OL cells (26, 35). In this case, HSV-1 egress is mediated by MVBs (36) and exosomes (37, 38). Finally, syncytial strains of HSV-1 can spread from cell to cell via syncytium formation, which occurs when infected cells fuse with neighboring uninfected cells (39).

HSV-1 and other alphaherpesviruses are well known to use cell-to-cell spread (40). HSV-1 spreads from one epithelial cell to another and then to the axons of sensory neurons, through cell-to-cell contacts, the major route of spread of HSV-1 in humans *in vivo*. HSV-1 virions remain mostly cell-associated, that is, they accumulate inside the cells and infect polarized cells or cells with numerous cell-to-cell contacts. That is a favorable type of viral diffusion, which allows HSV-1 particles to pass across epithelial and synaptic junctions while avoiding the effect of neutralizing antibodies (40). Therefore, the trafficking of virions to cell junctions might enhance viral spread and allow viruses to avoid host immune response (41, 42).

We previously showed that virions are associated with MAL-positive vesicles in the cell processes of OLs (25). Another member of the MAL proteolipid family, MAL2, has also previously been shown to be involved in HSV-1 infection (43). The present study confirmed that HSV-1 virions trafficked along cellular processes together with structures positive for MAL. These vesicles reached the tip of the processes that were contacting uninfected cells. Eventually, this targeting of virions led to infection of adjacent cells. Functionally, the depletion of MAL led to a significant decrease of plaque formation, indicating that MAL plays an important role in viral spread from OLs. Since extracellular infectious particles were not directly measured, the role of MAL in morphogenesis and egress will have to be clarified by further experiments. However, MAL depletion did not affect immediate early, early, and late gene expression, and visualization of cells infected with K26GFP showed the presence of viral replication without formation of plaques, suggesting that MAL depletion was affecting the late stages of the viral cycle. In addition, infection in the presence of pooled human serum—added to neutralize extracellular virus and restrict plaques to those formed by cell-to-cell spread—produced a decrease in the number of lytic plaques in MAL-silenced cells compared to nontarget controls, supporting the hypothesis of cell-to-cell spread involvement.

Previous reports have suggested a relevant role for MVBs in HSV-1 envelopment and egress (36, 44). Here, electron microscopy experiments revealed for the first time the presence of HSV-1 progeny virions enclosed in MAL-positive MVBs. Therefore, another mechanism affected by MAL protein might be the transport of virions through these structures. In the absence of MAL, the formation and traffic of MVBs in T cells is greatly impaired. In MAL-silenced T cells, tetraspanin-enriched microdomains are not incorporated efficiently into intraluminal vesicles of MVBs, leading to MAL’s accumulation on the limiting membrane of MVBs. These aberrant MVBs will be diverted to lysosomes for degradation (7, 8). Thus, it is reasonable to speculate that, in MAL-depleted OLs, MVBs containing virions might be degraded or diverted to aberrant pathways, thus disrupting the viral cycle.

Correct trafficking of HSV components during egress is a complex process that requires the coordinated activity of cellular and viral components. The critical participation of host factors such as the protein tyrosine phosphatase PTP1B in cell-to-cell spread has been recently reported (45). On the HSV side, the heterodimer of two glycoproteins, gE/gI, is critical to mediate cell-to-cell spread in epithelial and neuronal tissues (42). In polarized epithelial cells, proper targeting of virions to the basolateral domains is dependent on gE/gI (40, 46). It is known that gE/gI localizes to the TGN during early phases of infection and redistributes from the TGN to epithelial cell junctions later (47). Given the role of MAL in the transport of vesicles from the TGN, the possibility that MAL and gE/gI cooperate during egress would be an interesting hypothesis to test. Likewise, the role of MAL in the secretion of extracellular MVs containing virions—unaffected by neutralizing antibodies—will also have to be further investigated.

In conclusion, our study reveals for the first time a role for MAL protein in a viral cycle. MAL is essential for lytic plaque formation in OLs and plays a role in HSV-1 spread from these cells. Because MAL also plays a role in protein sorting and extracellular vesicle formation in epithelial cells, which are among the main targets of HSV-1, further studies will have to confirm whether MAL also plays a role in virus spread in epithelia.

## MATERIALS AND METHODS

### Antibodies and reagents.

Horseradish peroxidase-conjugated secondary anti-IgG antibodies were purchased from Millipore (Billerica, MA, USA). Alexa 488- and Alexa 647-conjugated secondary antibodies were obtained from Molecular Probes (Eugene, OR, USA). Polyclonal rabbit anti-HSV-1 antibody was from Dako. Living Colors anti-reef coral fluorescent protein (RCFP) polyclonal pan antibody was from Clontech. Anti-LAMP-1 mouse monoclonal antibody H4A3 was from DSHB (Developmental Studies Hybridoma Bank, University of Iowa, Iowa City, Iowa, USA). Sheep anti-TGN46 polyclonal antibody was from Serotec. Low-glucose Dulbecco modified Eagle (DMEM), fetal bovine serum (FBS), pooled human serum, carboxymethylcellulose sodium salt (CMC), and medium-viscosity and protease inhibitor cocktail were purchased from Sigma Chemical Co. (St. Louis, MO, USA). Mowiol was from Calbiochem (Merck Chemicals, Germany).

### Cell lines and viruses.

The human HOG cell line, established from a surgically removed human oligodendroglioma (48), was kindly provided by A. T. Campagnoni (University of California, UCLA, USA). This cell line was transfected with MAL-diHcRed—a construction consisting of MAL protein tagged with diHcRed, a dimeric red fluorescent protein—as described before (49). Cells were cultured on petri dishes in growth medium containing low-glucose DMEM supplemented with 10% fetal bovine serum (FBS), penicillin (50 U/ml), and streptomycin (50 μg/ml) at 37°C in an atmosphere of 5% CO_2_.

The HSV-1 K26GFP was a kind gift of P. Desai (Johns Hopkins University, Baltimore, USA). It was generated by fusing GFP to the HSV-1 capsid protein VP26 (31). Viruses were propagated and titrated on Vero cells. The virus HSV-1 (KOS) gL86, a β-galactosidase-expressing version of the KOS strain (34), was propagated in 79VB4 cells, a Vero-derived cell line stably expressing gL. 79VB4 cells and HSV-1 (KOS) gL86 were a kind gift of R. Longnecker (Northwestern University, Chicago, USA).

### Viral infections.

For viral infection assays, cells were mock infected or infected with the corresponding virus and maintained for viral adsorption at 37°C for 1 h in DMEM with antibiotics in the absence of fetal calf serum (FCS). Subsequently, cells were rinsed and cultured in DMEM with FCS 10%. Viral titer was quantified by an endpoint dilution assay determining the TCID_50_ in Vero cells, considering the final dilution that showed cytopathic effect and using the Reed and Muench method.

For the plaque assay, confluent monolayers of cells plated in 6-well tissue culture dishes were infected with HSV-1 at different MOIs. After viral adsorption, cells were washed and overlaid with CMC. The CMC solution was prepared in distilled water at 2% (wt/vol) and stirred at room temperature for 1 h. CMC overlay (1% final concentration) was prepared by mixing equal volumes of CMC 2% and 2× concentrated growth medium. To each well, 2 ml of CMC overlay were added. Plates were incubated at 37°C in a humidified 5% CO_2_ incubator for 48 h. Alternatively, pooled human serum 1% was added to the CMC overlay. After 48 h, this CMC overlay was aspirated; cells were washed with phosphate-buffered saline (PBS) and fixed in 4% paraformaldehyde for 20 min, and plaques were visualized by staining with crystal violet.

### Viral entry assay.

To determine HSV-1 entry, confluent monolayers of HOG-MAL-diHcRed cells plated in 96-well tissue culture dishes were infected with a recombinant HSV-1 (KOS) gL86, which expresses beta-galactosidase upon entry into cells. After 6 h p.i., beta-galactosidase assays were performed using a soluble substrate *o*-nitrophenyl-β-d-galactopyranoside (ONPG) assay. The enzymatic activity was measured at 410 nm using a Benchmark microplate reader (Bio Rad). HSV-1-resistant CHO-K1 cells were used as a control.

### Immunoblot analysis.

Samples were subjected to SDS-PAGE in 10% acrylamide gels under nonreducing conditions and transferred to Immobilon-P membranes (Millipore). After blocking with 5% nonfat dry milk and 0.05% Tween 20 in PBS, blots were incubated for 1 h at room temperature with primary antibody. After several washes with 0.05% Tween 20 in PBS, blots were incubated for 1 h with secondary antibody coupled to horseradish peroxidase, washed extensively, and developed using an enhanced chemiluminescence Western blotting kit (ECL; Amersham, Little Chalfont, UK).

### Immunofluorescence microscopy.

Cells grown on glass coverslips were fixed in 4% paraformaldehyde for 20 min and rinsed with PBS. Cells were then permeabilized with 0.2% Triton X-100, rinsed, and incubated for 30 min with 3% bovine serum albumin in PBS with 10% human serum, to block the HSV-1-induced IgG Fc receptors. For double- and triple-labeled immunofluorescence analysis, cells were incubated for 1 h at room temperature with the appropriate primary antibodies, and cells were then rinsed several times and incubated at room temperature for 30 min with the relevant fluorescent secondary antibodies. Controls to assess labeling specificity included omission of the primary antibodies. After thorough washing, coverslips were mounted in Mowiol. Images were obtained using an LSM 510 Meta system (Carl Zeiss) coupled to an inverted Axiovert 200 microscope. Processing of confocal images and colocalization analysis were made using Fiji-ImageJ software.

### Time-lapse fluorescence video microscopy.

For confocal living cell imaging, HOG MAL-diHcRed cells were cultured and infected on 35- mm glass-bottom culture dishes. The dynamics of viral infection were monitored in HOG MAL-diHcRed cells infected with the HSV-1 k26GFP virus. Video microscopy was performed on a confocal multispectral Leica TCS SP5 system (Centro Nacional de Biotecnología, CNB, Madrid, Spain) using a 63× objective. Cells were kept in an incubation system at 37°C in a humidified 5% CO_2_ atmosphere. Phase-contrast (DIC) and fluorescent images were acquired overnight every 15 min. Videos were obtained by merging the *z* axis projection of the confocal slices using LAS AF v.2.3.6 software (Leica Microsystems). Images were processed with Fiji software.

### Immunogold-labeling electron microscopy.

HOG MAL-diHcRed cells were mock infected or infected with HSV-1 at an MOI of 1 and incubated for 24 h at 37°C. Then cells were washed with PBS and fixed in 4% paraformaldehyde in 0.1 M sodium phosphate buffer, pH 7.4, at 4°C for 2 h. After that, cells were incubated overnight with 8% paraformaldehyde in 0.1 M sodium phosphate buffer, pH 7.4, at 4°C. Fixed cells were washed in PBS containing 20 mM glycine and embedded in 12% gelatin, infiltrated with 2.3 M sucrose, and frozen in liquid nitrogen. Cryosections were stained with a rabbit anti-RCFP antibody, which detects diHcRed, the red tag attached to exogenous MAL proteolipids. Primary antibody was detected with 15 nm anti-rabbit-gold (British BioCell, Cardiff, UK). Cryosections were examined with a JEM1010 transmission EM (Jeol, Tokyo, Japan).

### RNA interference-mediated silencing.

HOG cells were transfected as described (27) with plasmids expressing MAL-shRNAs. Briefly, 24 h prior to transfection of the HOG MAL-diHcRed cell line, 5 × 10^4^ cells were plated in 24-well tissue culture dishes with growth medium. Cells were transfected with 2 μg of DNA, using the JetPEI reagent according to the manufacturer’s instructions, and incubated with the DNA for 24 h in growth medium. Selection of stable cell transfectants was carried out 48 h after transfection in the presence of 2 μg/ml puromycin in growth medium, and MAL silencing was analyzed using immunoblots with the 6D9 mouse anti-MAL antibody (4). Plasmids encoding the nontarget control (pLKO.1-puro, 10121629MN) and MAL shRNAs TRCN0000117989 (shRNA-2) and TRCN0000117991 (shRNA-3) were from Sigma (MissionH TRC-Hs shRNA libraries, Sigma-Aldrich).

### Real-time quantitative RT-PCR assay.

A real-time quantitative RT-PCR assay was performed as previously described (50). Briefly, total RNA from triplicate samples of cells infected with HSV-1 cultured in 60-mm dishes was extracted using an RNeasy minikit (Qiagen, Valencia, CA, USA). RNAs were extracted at 3, 6, and 9 h p.i. to analyze ICP4, viral polymerase, and gC, respectively. RNA integrity was evaluated on an Agilent 2100 bioanalyzer (Agilent Technologies, Santa Clara, CA), and quantification of RNA was carried out on a Nanodrop ND-1000 spectrophotometer (Thermo Fisher Scientific). All the samples showed 260/280 ratio values of around 2, which correspond to pure RNA. Genomic DNA contamination was assessed with amplification of representative samples without reverse transcriptase (RT). Primer sequences were as follows: ICP4 (5′-ATGGGGTGGCTCCAGAAC-3′ forward and 5′-CTGCCGGTGATGAAGGAG-3′ reverse), viral polymerase (5′-CAGCAGATCCGCGTCTTTAC-3′ forward and 5′-GCAGAATAAAGCCCTTCTGGT-3′ reverse), and gC (5′-TGTAACTTCGACCCGCAAC-3′ forward and 5′-CGAGACAGACCGCAGTACAC-3′ reverse). RT-qPCR and data analysis were performed by the Genomics Core Facility at the Centro de Biología Molecular Severo Ochoa (CSIC-UAM). The NormFinder algorithm was used to identify GAPDH (glyceraldehyde-3-phosphate dehydrogenase) as the most suitable gene for the normalization due to its high stability. Oligonucleotides were from Sigma-Aldrich.

### Statistics.

Data are means ± standard deviation. Student’s *t*-tests were used to determine significant differences between groups (*, *P < *0.05).

## Supplementary Material

Supplemental file 1

Supplemental file 2

Supplemental file 3

Supplemental file 4
