# Supplementary material for: Herpes Simplex Virus 1 Spread in Oligodendrocytic Cells Is Highly Dependent on MAL Proteolipid
Source: J Virol. 2020 Jan 31;94(4):e01739-19. doi: 10.1128/JVI.01739-19 (PMC6997773; doi:10.1128/JVI.01739-19)
Supplement: Supplemental file 1 [file JVI.01739-19-s0004.pdf]

Supplemental Video Legends

S1. Colocalization of exogenous MAL in HSV-1 K26GFP infected cells. Cells plated in 35 mm glass-bottom dishes were infected with HSV-1 K26GFP at a m.o.i. of 1. Six hours later we performed video microscopy recording green (GFP, virus), red (exogenous MAL) and DIC signals. The video shows MAL-diHcRed accumulation in the poles of balloon infected cells. Gradually, virions gather near the cell poles and colocalize with MAL to finally traffic together along the processes.

S2. Traffic of exogenous MAL-associated virus from cell to cell. HOG-MAL-diHcRed cells plated in 35 mm glass-bottom dishes were infected with HSV-1 K26GFP at a m.o.i. of 1. Ten hours later we performed video microscopy recording green (GFP, virus), red (exogenous MAL) and DIC signals. The traffic of MAL from one cell to another through the processes is patent. After 5:45 h a cluster of MAL and virions has reached the neighbouring cell.

S3. Traffic of exogenous MAL-associated virus to uninfected cells. HOG-MAL-diHcRed cells plated in 35 mm glass-bottom dishes were infected with HSV-1 K26GFP at a m.o.i. of 1. Ten hours later we performed video microscopy recording green (GFP, virus), red (exogenous MAL) and DIC signals. The traffic of MAL, through the processes, from one infected cell to a cell lacking viral GFP signal is patent.
